# Safety Concerns of Traditional Chinese Medicine Injections Used in Chinese Children

**DOI:** 10.1155/2019/8310368

**Published:** 2019-06-23

**Authors:** Liping Tan, Mujin Li, Ying Lin

**Affiliations:** Medical College of Shaoguan University, Shaoguan, Guangdong 512026, China

## Abstract

**Objective:**

To explore risks underlying traditional Chinese medicine (TCM) injection-related adverse drug reactions (ADRs) in Chinese children, and to discuss the implications of postmarketing reevaluation studies.

**Methods:**

We identified potential cases of exposure to TCM injections for children (<18 years of age) and adults (18 years and upwards) from database of ADRs. First, the associations between TCM injection-related ADRs and three administration routes (i.e., intravenous or intramuscular administration, oral administration, and external use) and the imbalance of TCM injection-related ADRs between the paediatric and adult populations were tested using the Chi-square (*χ*^*2*^) test. Second, the proportional reporting ratio (PPR) was applied to identify statistically significant associations between drugs and anaphylactic shock in the paediatric population.

**Results:**

The *χ*^*2*^ test revealed that the highest frequency of paediatric ADRs was due to 5 types of herbal injections (i.e., Shuanghuanglian (SHL), Yuxingcao (YXC), Qingkailing (QKL), Xiyanping (XYP), and Reduning (RDN) herbal injections) (*P*<0.000), and the reports of ADRs attributed to the XYP and RDN herbal injections in children accounted for a greater proportion than the reports for adults (*P*<0.000). The PPR identified 5 types of herbal injections-anaphylactic shock pairs (i.e., the SHL, XYP, QKL, YXC, and Fufang Danshen herbal injections) that met the minimum criteria (i.e., a PPR of at least 2 and *χ*^*2*^ of at least 4 and three or more cases), which suggested that TCM injections were significantly associated with anaphylactic shock.

**Conclusions:**

TCM injections pose graver risks to the paediatric population than the adult population. To achieve optimal benefits and minimal risk to children treated with TCM injections, we suggest reevaluating the effectiveness and safety, monitoring the risks, and promoting rational use of TCM injections in Chinese children.

## 1. Introduction

Traditional Chinese medicine (TCM) has long been regarded as integral to the Chinese national essence. The development of TCM injections is considered to be a great achievement of TCM modernization. A TCM injection is a sterile preparation made with one or more purified extract of Chinese herbal drugs that is injected into the human body as a solution, emulsion, powder, or concentrated solution that is made into a solution before application [1]. However, there is an ongoing debate about the safety of the clinical use of TCM injections in light of the large number of adverse drug reaction (ADR) reports and literature in China.

Seventy-eight years ago, the Chaihu herbal injection was the first herbal injection to be developed and used in China. This injection played an important role in defervescence. However, the Chaihu herbal injection is known to produce anaphylactic shock and fatal anaphylaxis, especially in children [2]. Therefore, the China Food and Drug Administration (CFDA) issued a notice contraindicating its use in children [3]. In addition to the Chaihu herbal injection, the CFDA has issued a series of public warnings and modified the instructions for use of nine other TCM injections used in children since 2006 (summarized in Table 1).

The toxicity of TCM injections has been a source of concern, since they can induce adverse drug reactions (ADRs), including allergic reactions such as anaphylactic shock, as a common side effect. As noted by Ji K et al., nine TCM injections used to treat common colds and upper respiratory tract infections caused serious anaphylaxis, and therefore he suggested improving the clinical safety of TCM injections used as medical treatments [4]. Guo YJ et al. also noted that anaphylactic shock caused by TCM injections for the treatment of cardiovascular and cerebrovascular diseases was not only common but also sometimes fatal [5]. Furthermore, a retrospective analysis based on the literature showed that the incidence of anaphylactic shock and lethal anaphylaxis caused by TCM injections was significantly higher than that of other TCM administration routes [6]. For this reason, TCM injections should be strictly monitored and carefully utilized.

Drug absorption, distribution, metabolism, and excretion during childhood apparently differ from those processes in adults. Moreover, the continuous development of children results in states not only differs from adults but also differs vastly within their own age groups. Children place higher demands on drugs than adults to achieve the optimal benefit and minimal risk. For these reasons, there is an urgent need to pay more attention to the clinical safety and promote the rational use of TCM injections in the paediatric populations. However, studies on TCM injections have tended to focus on adults rather than on children. Insufficient attention has been paid to the potential risks associated with TCM injections used in children and the imbalance of TCM injection-related ADRs between the paediatric and adult populations. Therefore, this study aimed to explore the characteristics and potential risks underlying TCM injection-related ADRs and to discuss the implications of the risks through studies of TCM injections. We hope that this research will contribute to a deeper understanding of the graver risks associated with TCM injections for the paediatric population and the importance of translating TCM from an experience-based to an evidence-based medicine system.

## 2. Methods

### 2.1. Data Source

The data that support the findings of this study are available from the National Scientific Data Sharing Platform for Population and Health (http://www.ncmi.cn/column/INDEX) which comprises basic medical, clinical medicine, public health, traditional Chinese medicine, pharmacy database, and population and reproductive health databases. In this study, ADR reports were collected from the pharmacy database. The data structure of the ADR reports consists of patient information (excluding the patient's name), administrative information, drug information, adverse events (AEs), patient outcomes, therapy measures, and indications for use/diagnosis. Key variables in this study included the patient's age, drug names, and ADR types.

### 2.2. Data Process

#### 2.2.1. Improving the Data Quality

Prior to data analysis, reports with missing ages were excluded, because age was the main variable in our study. Because the drug names were not standardized, a harmonization procedure was implemented using the CFDA data [7]. Briefly, this procedure consisted of unifying the brand names into the generic drug names and correcting mistakes in recording drug names. Following improvement of the data quality, we kept only the suspected and the concomitant drugs and deleted drug-drug interactions. Suspected drugs refer to drugs suspected as the cause of ADRs, and concomitant drugs refer to drugs used concurrently that were not suspected by the reporter. Only medicinal products reported as ‘suspect' or ‘concomitant' are taken into account in spontaneous reporting systems in China, and no drug-drug interactions are considered [8]. Finally, reports with medication errors were excluded from our analysis.

#### 2.2.2. Stratifying the Patients by Age

An ADR/adverse event (AE) database specific for children has not been established in China. In this study, the raw data included not only paediatric reports but also adult reports. Therefore, the raw data may not effectively reveal associations between medicinal products and AEs when the drug use is age-specific or when an age-specific risk is suspected. For the purpose of stratification by age, only reports of ADRs occurring in children (<18 years of age) were retained, resulting in a reduction in the number of reports from 7366 to 701. After stratifying the patients by age, we took the following steps: (i) paediatric reports involving TCM injections were extracted from the 701 reports of ADRs using the search function to detect potential risks of TCM injections in children, and (ii) adult reports (18 years and upwards) of TCM injection-related ADRs were collected to evaluate the imbalance between the paediatric and adult populations.

### 2.3. Statistical Analysis

Signals of disproportionate reporting (SDRs) refer to statistical associations between medicinal products and ADRs (i.e., drug-ADR pairs). Different statistical methods are used to generate SDRs for detection. The proportional reporting ratio (PRR) was used to detect SDRs in our study. SDRs for the investigated drugs were identified by calculating the value of the PRR for all suspected TCM injection-ADR pairs. The criteria applied to define a signal of disproportionate reporting are as follows: (i) the PRR value ≥2, (ii) the Chi-square (2) value ≥4, and (iii) the number of individual cases greater than or equal to 3. The PRR is A/(A+B) divided by C/(C+D) in a two-by-two table (see Table 2).

*χ* ^2^ is used as an alternative measure of associations between the investigated drugs and ADRs based on the following calculation:

(1) $$\begin{array}{c} \chi^{2}=\frac{{\left(AD-BC\right)}^{2}\left(A+B+C+D\right)}{\left(A+B\right)\left(C+D\right)\left(A+C\right)\left(B+D\right)} \end{array}$$

Further details of the PRR used in signal detection can be found in the European Medicines Agency guidelines [9].

The statistical analyses were performed using the SPSS for Windows version 19.0 software (IBM, Somers, NY, USA). The *χ*^*2*^ test was performed to analyse the data, and differences with* P*<0.05 were deemed statistically significant.

All data used in the study are aggregate data; no individual case reports or identifiable patient data were used. Hence, according to applicable legislation, no approval from the ethics review board was needed for the study.

## 3. Results

A total of 317 reports involving children and adults could be used in the analysis, which included 16 types of herbal injections (see Table 3), 16 types of oral Chinese medicines, and 4 types of TCM for external use. A total of 102 reports of ADRs induced by TCM were associated with children and adolescents aged less than 18 years (see Table 4).

**Figure 1:**
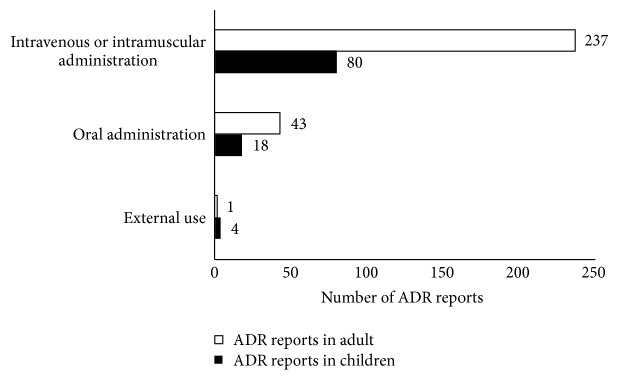
The number of ADR reports in the paediatric and adult population treated with 36 types of TCM via three administration routes. The ADR reports of the paediatric and adult population are compared, which are induced by 36 types of TCM via three administration routes (i.e., intravenous and intramuscular injection, oral administration and external use). TCM: traditional Chinese medicine; ADR: adverse drug reaction.

Figure 1 presents an overview of the ADR reports in the paediatric and adult populations treated with the 36 types of TCM via three administration routes (i.e., intravenous or intramuscular injection, oral administration, and external use). This figure shows the high incidence of ADRs induced by intravenous or intramuscular injection. The highest frequency of paediatric and adult ADRs was found to be due to intravenous or intramuscular injection, accounting for 80 (78.4%) and 237 (84.3%) cases, respectively. The subsequent statistical analysis was focused on whether a significant difference existed in reports of ADRs when children were treated with TCM via the three administration routes. The *χ*^*2*^ test (one sample) shown in Table 4 revealed a significant difference in ADR reports when the paediatric population was treated with TCM via the three administration routes (*P*<0.000). TCM injections are injected into the human body via intravenous, intramuscular, local, and acupoint injection, although the overwhelming majority of TCM injections are performed via intravenous or intramuscular injection [1]. These results can be expected that any kind of injections (i.e., TCM injections, biopharmaceuticals and chemicals) would be more dangerous than oral or external use.

**Figure 2:**
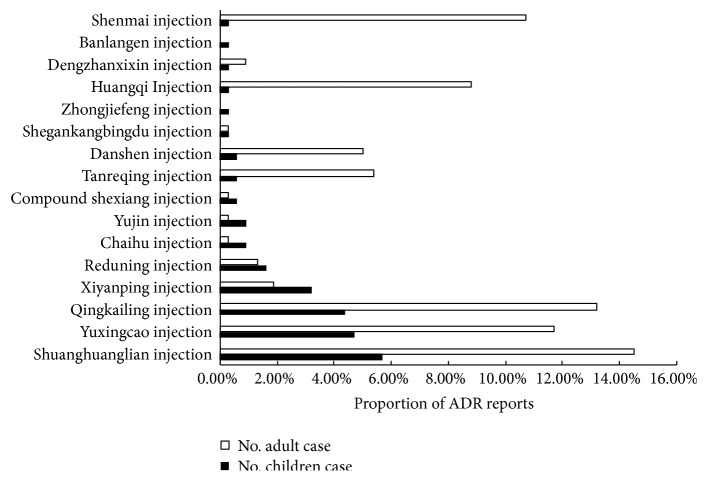
The proportion of ADR reports in the paediatric and adult population treated with 16 types of TCM injections. The reports of ADRs attributed to 16 types of TCM injections are compared between the paediatric and adult population. TCM: traditional Chinese medicine; ADRs: adverse drug reactions.

The next section of the statistical analysis was concerned with the imbalance between paediatric and adult reports of ADRs attributed to the 16 types of TCM injections. The frequency distribution and the reporting proportions of the 317 reports of ADRs caused by the 16 types of TCM injections are shown in Table 3. Although the 16 types of TCM injections were common in both children and adults, the frequency distribution of reports for individual drugs varied.  Figure 2 showed that the proportion of ADR reports attributed to five TCM injections (i.e., the Shuanghuanglian (SHL), Yuxingcao (YXC), Qingkailing (QKL), Xiyanping (XYP), and Reduning (RDN) herbal injections) were ranked in top five, and the ADRs of three injections (i.e., the SHL, YXC, and QKL herbal injections) happened highly both in children and adults. Notably, the reports of ADRs attributed to the XYP and RDN herbal injections in children accounted for a greater proportion than the reports for adults. The *χ*^2^ test was conducted to examine whether a significant difference existed between the paediatric and adult reports of ADRs caused by the 16 types of TCM injections. The Pearson *χ*^2^ test must meet two criteria: (i) all expected frequency values (T)≥1, and (ii) the proportion of T (1≤T<5) does not exceed 20%. In our study, the proportion of T (T<5) was 56.3%, and the minimum T was 0.25; thus, Fisher's precise inspection (Monte Carlo method) was used to analyse the data. As shown in Table 3, a significant difference (*P*<0.000) was found between the paediatric and adult reports of ADRs. Overall, these results indicated that (i) the SHL, YXC, QKL, XYP, and RDN injections should be used with caution in children because they showed the highest incidence of ADRs, especially their incidences of ADRs were also high in adults, and (ii) the reports of ADRs attributed to the XYP and RDN herbal injections in children accounted for a greater proportion than the reports for adults, partly because they might be more widely used in paediatric clinical application than other herbal injections.

In the final part of the study, we searched for all reports associated with anaphylactic shock using the keyword search function of the database. The total number of cases that reported for anaphylactic shock and the PPR and *χ*^*2*^ values is shown in Table 5. A total of 66 cases reported for anaphylactic shock, of which 9 reports of anaphylactic shock were caused by five types of TCM injections (i.e., the SHL, XYP, QKL, Fufang Danshen, and YXC herbal injections). In our study, the statistical method identified the TCM injection-anaphylactic shock pairs that met the minimum criteria (i.e., a PPR of at least 2, *χ*^*2*^ of at least 4 and three or more cases). The statistical metrics suggested that TCM injections were significantly associated with anaphylactic shock. As shown in Table 6, the SHL and XYP herbal injections resulted in anaphylactic shock in the paediatric population.

## 4. Discussion

The initial objectives of our study were to explore the characteristics and risks underlying TCM injection-related ADRs in the paediatric population. Concerning the question of the imbalance of TCM injection-related ADRs between the paediatric and adult populations, the results showed that the paediatric reports for the XYP and RDN herbal injections accounted for a greater proportion than the reports for adults. The most obvious finding that emerged from the analysis was that the proportion of ADR reports attributed to 5 types of herbal injections (i.e., the SHL, YXC, QKL, XYP, and RDN herbal injections) were ranked in top five. The expected result was that TCM injections were more likely to cause ADRs in children than oral or external use. Another important finding was that a significant correlation existed between TCM injections and anaphylactic shock.

### 4.1. Implications of the Risk

**Figure 3:**
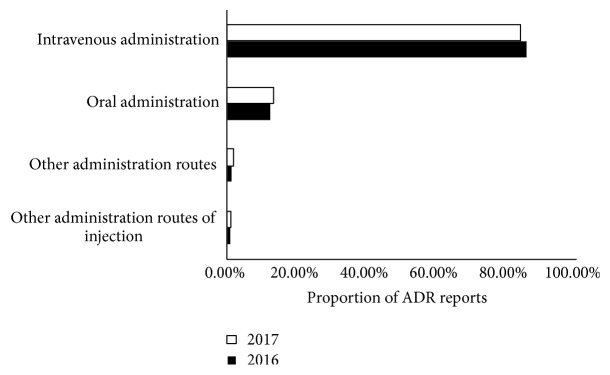
Distribution of reports of serious ADRs/AEs induced by TCM via four administration routes. The 2016 and 2017 annual reports on ADRs from the CFDA are compared to find the different distribution of serious ADRs/AEs induced by TCM via intravenous administration, other injection routes, oral administration, and other administration routes of injection. ADRs: adverse drug reactions, AEs: adverse events, TCM: traditional Chinese medicine, CFDA: China Food and Drug Administration, ADR: adverse drug reaction.

In our study, the frequency reports of ADRs for intravenous or intramuscular injection were highest, accounting for 80 cases (78.4%) during paediatric use of TCM. Similar results were found in annual reports on ADRs issued by the CFDA in 2016 [10] and 2017 [11]. The distributions of reports of serious ADRs/AEs induced by the four TCM administration routes are shown in Figure 3. This figure shows the high incidence of ADRs/AEs induced by TCM delivered via the intravenous administration route. Although the retrospective analysis targeted adults rather than children, similar attitudes were expressed by Lin M and Ji K, who were concerned that TCM injections might be more dangerous in the clinic than other drugs [4, 6]. Regardless of TCM, chemicals or biopharmaceuticals, it can be expected that any kind of injections would be more dangerous than oral or external use. Because of disadvantages of injections, the technical requirements and quality standards of injections are very strict, which require that any kind of injections must be of clear ingredients, high purity, proven efficacy, and clear mechanisms of toxic and side effects. The consensus on the technical requirements and quality standards of injections are reached in the field of biopharmaceuticals and chemicals. However, TCM injections do not reach all of the required standards, so they might be more dangerous than biopharmaceuticals and chemicals, especially when drugs are used in children.

Among all of the ADRs attributed to TCM injections in the literature, allergic reactions had the highest incidence, including anaphylactic shock and fatal anaphylaxis [12, 13]. Another study showed that severe anaphylaxis and anaphylactic shock caused by TCM injections accounted for 82.2% and 86.9% of the total cases of severe anaphylaxis and anaphylactic shock caused by TCM, respectively [6]. However, these researches failed to focus on children, and the results were not based upon statistical methods to detect the associations between ADRs and TCM. In this study, the PPR and *χ*^*2*^ values were used to determine whether the paediatric ADRs attributed to TCM injections were reported more than those for other drugs. Although the approach does not evaluate the causality or the exact incidence of the reaction in the paediatric population, the PPR and *χ*^*2*^ values are measures related to the strength of the association (the higher the PRR, the greater the strength of the signal) and suggest whether a detailed evaluation and further investigation are needed for specific drugs [14]. In our study, the statistical methods verified that five TCM injection-anaphylactic shock pairs met the minimum criteria (i.e., a PPR of at least 2, *χ*^*2*^ of at least 4 and three or more cases). The statistical metrics suggested that TCM injections were statistically associated with anaphylactic shock. This finding, although preliminary, suggested that a detailed evaluation and further investigation of TCM injections were indispensable, especially in the paediatric population. The SHL and XYP herbal injections resulted in anaphylactic shock in the paediatric population. Fortunately, the SHL herbal injection was contraindicated in children under four years because of serious ADRs by the CFDA. However, the health risk of giving children the XYP injection has not received sufficient attention from healthcare professionals and regulatory authorities.

Regarding the imbalance in ADR reporting between children and adults, our finding was consistent with that of Blake KV et al., who confirmed that paediatric ADRs were more common than those in adults in terms of the reactions and drugs involved [15]. In our study, the top five reports of ADRs were obtained for the SHL, QKL, YXC, XYP, and RDN herbal injections, and the paediatric reports of the RDN and XYP herbal injections accounted for a greater proportion of reports than those for adults. As shown in Table 1, this was a mixed blessing for paediatric patients: the YXC herbal injections were contraindicated in children of all ages by the CFDA, but the SHL and QKL herbal injections were only contraindicated in children during the ages of 0~4. Furthermore, the RDN and XYP herbal injections are still used within paediatric patients [16–18]. In the literature review, associations between these two herbal injections and paediatric ADRs were found. Clinical data from 1180 paediatric patients treated with the XYP herbal injection from January 2011 to December 2015 were analysed and showed that an age less than 3 years, intravenous drip, and combination use of drugs (3 types) were the main risk factors for ADRs due to the XYP herbal injection [19]. In 2011, 1048 reports of ADRs involving children under 14 years old were connected with the XYP herbal injection, accounting for 71% of the total reports [20]. Hence, the National Center for ADR Monitoring, China, posted bulletins that warned of the potential for severe anaphylaxis due to the XYP herbal injection, especially in children. A total of 125 patients suffered from ADRs/AEs caused by the RDN herbal injection in Chongqing, China, between January 2010 and June 2013, and the occurrence was higher in children under 2 years old (48.0%) [21]. The series of incidents summarized above highlights the graver risks associated with the use of these herbal injections in Chinese children compared to that of adults, especially when drug was used in younger children. Therefore, there is an urgent need to monitor the risks and promote the rational use of TCM injections in the paediatric populations.

According to the findings of this study, five herbal injections (i.e., the SHL, QKL, YXC, XYP, and RDN herbal injections) should be used with caution because they showed the highest numbers of total events, among which two herbal injections posed graver risks for the paediatric population than for the adult population. However, there was only one of five herbal injections which was contraindicated in children of all ages by the CFDA. How to achieve optimal benefits and minimal risk in children treated with TCM injections is an important issue for future research, supervision, and use.

### 4.2. Research Implications

#### 4.2.1. Clear Drug Instructions on the Use for Children

Physicians require evidence-based information on the efficacy and risk of a medicinal product when prescribing medicines. Patients, parents, and caregivers need understandable descriptions of the benefits, precautions, and risks in order to consent to take/administer a medicine. Pharmacists need evidence-based information to adequately dispense the medicine and provide user instructions to the patient/parent. Nurses require evidence-based information to enable safe preparation and administration of medicines. The knowledge of health professionals and patients mainly is based on drug instructions, which are official documents guiding health professionals and patients through rational drug use. However, insufficient use information is availed for children in the drug instructions of the RDN and XYP herbal injections. As shown in Table 7, two types of valuable information (i.e., drug interactions and usage of medication in the paediatric populations) are deficient. This lack may be one reason for the high incidence of ADRs attributed to the RDN and XYP herbal injections when used within the paediatric population. In view of ADRs caused by TCM injections, the CFDA put forward correlated suggestions to doctors/practitioner on the clinical application in children. What the CFDA would strongly suggest are (i) to detailedly check the patient's allergy history before using TCM injections, (ii) to prudently administer drug combination, (iii) to contraindicate a mixture of TCM injections and other medicines, (iv) to strictly follow the proper dosage on drug instructions, and (v) to strengthen medication monitoring during drug use [20]. At the meantime, the pharmaceutical manufacturers should take the responsibility for perfecting medical information for age-specific patients. Aim at the imperfection of the drug instructions of TCM injections, Wu, S. X et al. suggested that postmarketing research of TCM injections should include clinical study on safety and efficacy and supplementary data on pharmacology, toxicology, drug interaction, and drug incompatibility [22]. Ji K et al. have strongly suggested that more accurate instructions for Chinese herbal injections should be formulated according to the clinical results to avoid misuse and avert potential future tragedies [4].

#### 4.2.2. Reevaluation on Postmarketing TCM Injections

Many physicians and scientists grant that TCM herbs may include useful molecules (e.g., one notable product that has emerged from TCM is artemisinin) but worry that TCM can be potentially test dangerous [23]. Some observations and experimental results have established aristolochic acid (AA), which is an ingredient in many TCM remedies, as a mutagen responsible for kidney failure and cancer [24–27]. Recently, whole-exome sequence data from patients with hepatocellular carcinoma, renal cell carcinoma, intrahepatic cholangiocarcinoma, and bladder cancer have suggested that AA-induced mutations may also contribute to the initiation and/or progression of these cancers [26]. Modern phytochemical studies have shown that each herb contains dozens or even hundreds of pure compounds. However, the majority of these compounds are unidentified compounds and/or have unconfirmed effects. Therefore, the assumption that many herbs may contain toxic or carcinogenic substances that are not recognized due to limitations of detection techniques and the latency period between exposure and the onset of symptomatic disease is prudent. Moreover, genetic determinants confer susceptibility to only ~5% of those exposed to some herbs [26]. For these reasons, we think that TCM injections should receive more attention concerning the potential risks of the unidentified chemical agents and unconfirmed effects. The premarket safety evaluation of drugs is the first protective barrier against the health/safety hazards. Due to historical reasons, TCM injections lacked of standardized premarketing research in their nascent stages; therefore postmarketing reevaluation was very important for evaluating of safety and efficacy of TCM injections. The necessity of postmarketing reevaluation studies on any drug, including TCM injections, is also determined by the disadvantages of premarketing research, such as simple research purpose, stringent medication conditions, short follow-up periods, special populations (paediatric population, elderly population, pregnant or breast-feeding women, etc.) excluded from subjects, etc. [28]. Safety and effectiveness are equally important for TCM, while the postmarketing reevaluation studies on TCM injections should be more concerned about safety aspects because TCM injections are more dangerous than oral or external use.

In recent years, more and more studied aimed at uncovering stricter and accurate evidence on the safety of TCM injections because the incidences of ADRs/AEs have gradually increased. The main contents of safety reevaluation emphasized by researchers are summarized as follows: risk assessment and management, early warning of ADRs, judgement and processing methods on ADRs, mechanisms of ADRs, detecting and screening allergens for injections in TCM, and data analysis based on Hospital Information System (HIS) [28].

The postmarketing clinical research provides the basis for the reevaluation of TCM injections. Using data from the HIS, reevaluation studies of the postmarketing safety of several different kinds of TCM injections were carried out by employing prospective nested case-control and prescription sequence analysis designs [29–31]. Wang LX et al. adopted this method to revalue the safety of the Shenqi Fuzheng injection by monitoring 30026 cases from 35 hospitals, and the result showed that incidence rate of ADRs was 0.17% [31]. These researches were the postmarketing ADRs/AEs study of TCM injections with large scale and multicenter assessment and can provide evidence-based information for safe clinical use of TCM injections.

Due to the high incidence of anaphylaxis, the investigation of sensitization of TCM injections has been a major challenge in TCM research areas [6, 32]. Selection of appropriate methods according to the characteristics of TCM injections is a key point. Network pharmacology is a compelling approach that offers a way to think about drug discovery, improve clinical efficacy and understand the side effects and toxicity of drugs [33]. The holistic philosophy of TCM shares much with the key ideas of network pharmacology, which considers multitarget strategies over single-target approaches [34]. Therefore, network pharmacology technology was used to construct the network model of TCM allergens, which identified that (i) honeysuckle, scutellaria, forsythia, and gardenia could easily induce clinical sensitization, (ii) gardenia, houttuynia, and isatidis might be herbals with a high risk of inducing anaphylactic shock, and (iii) beta-sitosterol, chlorogenic acid, and palmitic acid of TCM were highly correlated with sensitization [6]. Network pharmacology provides the possibility of screening for potentially sensitizing components and risks factors in TCM injections in a high-throughput manner.

To provide safe, effective, and inexpensive medicines for the public, draft guidelines on the quality control and technical requirements for safety reevaluation of TCM injections were adopt by the State Food and Drug Administration in 2009 [35] and draft guidelines on relative techniques in 2010 [36]. However, regulations on the postmarketing reevaluation and relative technical guidelines (especially TCM) are imperfect, as a result, respective responsibilities of government departments, enterprises, and research institutions are unclear, and the reevaluation procedures and contents are not clearly defined [28]. It is noteworthy that the above factors may produce strong adverse effects to reevaluation on postmarketing TCM injections. Xie YM et al. suggested to establish new and whole regulations, which should cover the following aspects: the risk assessment and management, ADRs monitoring and reporting, ADRs relief, drug recall management, and regulations for the reevaluation of TCM (including injections), as well as the corresponding technical specifications and guidelines [28].

### 4.3. Limitations

Spontaneous reporting of ADRs has been shown to be an important method for reassessment of the risk-benefit balance in patients. This method can be considered particularly important in children, since drugs are not routinely tested in the paediatric population [37]. Nevertheless, data from the spontaneous reporting system in China are not open but instead are exclusive. Lack of access to data is a major barrier to research on the safety of drugs in children. On January 5, 2017, the data from the National Scientific Data Sharing Platform for Population and Health were publicly released, which meant that a large amount of Chinese health data was made available to researchers. At present, this database is still in the initial stage and is not perfected; for example, the number of ADRs is small, and the data need to be updated. From this point of view, a gap still exists between this data set and real world big data.

Importantly, the limitations of spontaneous reporting should be taken into account in the interpretation of the results of the present study. Underreporting and low quality of data are the defects of ADR reports [38]. In the present study, we had to delete some data from our study, because much valuable information for ADRs was missing (e.g., the dosage, demographic characteristic of the patients and administration route).

## 5. Conclusions

In the present study, we explored the characteristics and risks underlying TCM injection-related ADRs in the paediatric population. We found that TCM injections pose graver risks to the paediatric population than to the adult population. Recently, the governing authorities have issued warnings of the clinical risk of TCM injections one after another, but these measures cannot fundamentally solve the key problems. For example, sufficient use information for children is not available from the drug instructions for TCM injections, which results in skepticism regarding their efficacy and safety from professionals and/or patients. To achieve optimal benefit and minimal risk for children treated with TCM injections, the effectiveness and safety must be reassessed, the risks must be monitored, and rational use must be promoted for TCM injections in the paediatric populations. We especially underscore careful application of TCM injections in the paediatric population.

## Figures and Tables

**Table 1:** The instructions of ten TCM injections modified by the CFDA since 2006.

Time	TCM Injections	Components	Instruments modified by the CFDA
Jun. 2018	Shuanghuanglian herbal injecton	1. Lonicerae Japonicae Flos 2. Scutellariae Radix 3. Forsythiae Fructus	contraindication in children under four years old and pregnant women
Jun. 2018	Qingkailing herbal injection	1. Cholic Acid 2. Hyodesoxycholic Acid from Pig Bile 3. Bubali Cornu 4. Baicalin 5. Margaritifera Concha 6. Gardeniae Fructus 7. Isatidis Radix 8. Lonicerae Japonicae Flos	contraindication in newborn, infant and pregnant women
May. 2018	Chaihu herbal injection	Bupleuri Radix	contraindication in children
Aug. 2016	Yinzhihuang herbal injection	1. Artemisiae Scopariae Herba 2. Gardeniae Fructus 3. Baicalin 4. Lonicerae Japonicae Flos	contraindication in neonates and infants
Oct. 2015	Guanxinning herbal injection	1. Salviae Miltiorrhizae Radix et Rhizoma 2. Chuanxiong Rhizoma	contraindication in children and pregnant women
Aug. 2012	Honghua herbal injection	Carthami Flos	contraindication in children and women during pregnancy or lactation
Mar. 2007	Fufang pugongying herbal injection	1. Taraxaci Herba 2. Houttuyniae Herba 3. Chrysanthemi Indici Flos	contraindication in children and pregnant women
Mar. 2007	Yujin herbal injection	1. Houttuyniae Herba 2. Lonicerae Japonicae Flos	Lack of the clinical data for use in children and pregnant women
Nov. 2006	Lianbizhi herbal injection	Andrographolide sodium bisulfite	Usage in children, pregnant women, lactating women and old people with caution
Nov. 2006	Yuxingcao herbal injection	Houttuyniae Herba	contraindication in children and pregnant women

TCM: traditional Chinese medicine

CFDA: China Food and Drug Administration

**Table 2:** Calculation of the PRR.

	Reaction(s) of interest	All other reactions
Drug of interest	A	B
All other drugs in database	C	D

PRR: proportional reporting ratio

**Table 3:** The ADR reports of the paediatric and adult population treated with 16 types of TCM injections.

TCM injections	ADR Reports		Chi-squared test
	Children (%)	Adult (%)	
Shegankangbingdu herbal injection	1 (0.3)	1 (0.3)	*P<*0.000
Shuanghuanglian herbal injection	18 (5.7)	46 (14.5)	
Compound shexiang herbal injection	2 (0.6)	1 (0.3)	
Qingkailing herbal injection	14 (4.4)	42 (13.2)	
Zhongjiefeng herbal injection	1 (0.3)	0 (0.0)	
Huangqi herbal Injection	1 (0.3)	28 (8.8)	
Dengzhanxixin herbal injection	1 (0.3)	3 (0.9)	
Chaihu herbal injection	3 (0.9)	1 (0.3)	
Banlangen herbal injection	1 (0.3)	0 (0.0)	
Shenmai herbal injection	1 (0.3)	34 (10.7)	
Yuxingcao herbal injection	15 (4.7)	37 (11.7)	
Xiyanping herbal injection	10 (3.2)	6 (1.9)	
Yujin herbal injection	3 (0.9)	1 (0.3)	
Reduning herbal injection	5 (1.6)	4 (1.3)	
Tanreqing herbal injection	2 (0.6)	17 (5.4)	
Danshen herbal injection	2 (0.6)	16 (5.0)	
Total	80 (25.2)	237 (74.8)	

Proportion of ADR reports of each drug was calculated by using the total number of ADRs of all drug as denominators. The Pearson Chi-squared test must meet two criteria: (i) all expected frequency values (T)≥1, and (ii) the proportion of T (1≤T<5) does not exceed 20%. In our study, the proportion of T (T<5) was 56.3%, and the minimum T was 0.25; thus, Fisher's precise inspection (Monte Carlo method) was used to analyse the data. Differences with *P<*0.05 were deemed statistically significant.

TCM: traditional Chinese medicine, ADR: adverse drug reaction

**Table 4:** The ADR reports of the paediatric population treated with 37 types of TCM via three administration routes.

Administration route	No. children case	Chi-squared test
Intravenous or intramuscular injection	80	*P*<0.000
Oral administration	18	
External use	4	
Total	102	

Differences with P<0.05 were deemed statistically significant.

TCM: traditional Chinese medicine, ADR: adverse drug reaction

**Table 5:** The PPR calculation-TCM injections and anaphylactic shock.

	Anaphylactic shock	All other reactions	Total	PPR	*χ* ^2^
TCM injections	9	186	195	5.8	31.2
All other drugs in database	57	7114	7171		
Total	66	7300			

TCM: traditional Chinese medicine, PRR: proportional reporting ratio

*χ* ^2^ = (AD − BC)^2^(A + B + C + D)/(A + B)(C + D)(A + C)(B + D)

PPR = A/(A + B)/C/(C + D)

**Table 6:** The reports of anaphylactic shock induced by TCM injections.

TCM injections	Adult cases	Children cases
SHL herbal injection	2	1
Fufang Danshen herbal injection	1	0
XYP herbal injection	2	2
YXC herbal injection	3	0
QKL herbal injection	1	0

TCM: traditional Chinese medicine, SHL: Shuanghuanglian, XYP: Xiyanping, YXC:Yuxingcao, QKL: Qingkailing

**Table 7:** The use information in the drug instructions of the RDN and XYP herbal injections.

TCM injections	Constituents	Indication	Dosage for children	Drug interactions	Usage of medication in children
RDN herbal injection	1. Artemisiae Annuae Herba 2. Lonicerae Japonicae Flos 3. Gardeniae Fructus	1. clearing heat 2. dispelling wind 3. detoxicating	Children ages 3 to 17	–	–
XYP herbal injection	Andrographolide Sulfonate	1. disappear hot 2. detoxify 3. stop dysentery	Children ages 1 to 17	–	–

RDN: Reduning, XYP: Xiyanping

“–”: No information

## Data Availability

The data used to support the findings of this study are available from the National Scientific Data Sharing Platform for Population and Health (http://www.ncmi.cn/column/INDEX).
